# Numerical Simulation for the Unsteady MHD Flow and Heat Transfer of Couple Stress Fluid over a Rotating Disk

**DOI:** 10.1371/journal.pone.0095423

**Published:** 2014-05-16

**Authors:** Najeeb Alam Khan, Shahnila Aziz, Nadeem Alam Khan

**Affiliations:** 1 Department of Mathematical Sciences, University of Karachi, Karachi, Pakistan; University of Adelaide, Australia

## Abstract

The present work is devoted to study the numerical simulation for unsteady MHD flow and heat transfer of a couple stress fluid over a rotating disk. A similarity transformation is employed to reduce the time dependent system of nonlinear partial differential equations (PDEs) to ordinary differential equations (ODEs). The Runge-Kutta method and shooting technique are employed for finding the numerical solution of the governing system. The influences of governing parameters viz. unsteadiness parameter, couple stress and various physical parameters on velocity, temperature and pressure profiles are analyzed graphically and discussed in detail.

## Introduction

The flow problems of non-Newtonian fluids are attracted the interest of many researchers because of its significance in modern technology and industries. The natural and industrial applications of such fluids are volcanic lava, extrusion of polymer fluids, drilling mud, suspension solutions, cosmetic and food products, solidification of liquid crystals, cooling of metallic plates in a bath, exotic lubricants, colloidal and many others. In recent times, among various non-Newtonian fluid models, the couple stress fluid model has got the special status because of the spin field in the fluid which sets up an anti-symmetric stresses, known as couple stresses. The basic theory and constitutive equations for couple stress fluid initially developed by Stokes [1], is one amongst the polar fluid theories which consider couple stresses in addition to the classical Cauchy stresses. This type of fluid encloses inflexible and random-oriented particles in a viscous medium. Examples of couple stress fluids are liquid crystals, colloidal fluids, liquids containing long-chain molecules such as polymer suspensions, blood, lubrications and additive electro-rheological fluids etc. These fluids are considered by many investigators because of the simplest model of couple stress fluid amongst others. (See recent attempts [2]–[4]).

Magnetohydrodynamics (MHD) occurs when a conducting fluid flows in the presence of an electromagnetic field, such that the induced electromagnetic force affects the motion of the fluid. Some significant technological applications of MHD are, in experiments of controlled thermonuclear fusion where a strong magnetic field is used to confine rings or columns of hot plasma, to generate electricity where liquid metals are driven through a magnetic field, etc. Moreover, MHD principles are used in plasma accelerators for ion thrusters, for spacecraft propulsion and for light-ion-beam powered inertial confinement. Some remarkable attempts have been made to study the influence of MHD on various flow situations. The influences of MHD stagnation point flow was analyzed by Hayat et al. [5], MHD flow in a circular magnetic field was considered by Kumar et al. [6], viscous dissipation and Joule heating on unsteady mixed convection MHD flow was discussed by Osalusi et al. [7]. Investigation of fractional MHD Oldroyd-B fluid over an oscillating plate was the subject of Jamil et al. [8], and the influence of MHD in the porous medium and heat transfer analysis of couple stress fluid between two parallel plates with variable viscosity was investigated by Sreenadh et al. [9]. Numerical methods to obtain the solutions of some complicated MHD flow problems were presented in [10]–[12].

Rotating-disk flow of non-Newtonian fluids is intriguingly growing interest, as it is momentous in the study of engineering flows on rotating machineries, spin-coating, centrifugal pumps, rotational viscometers, computer storage devices and some aerodynamic related problems in fluid mechanics. Taking into account both industrial and technological applications, there have been a number of studies presented in literature on steady flow of various types of fluid over a rotating disks and sphere with and without considering magnetic effect [13]–[25]. It is worth mentioning that the real world applications encounter the unsteadiness in the flow and temperature fields of almost all processes. Osalusi et al. [26] presented the effects of Ohmic heating and viscous dissipation on unsteady MHD and slip flow over a porous rotating disk. The series solutions for unsteady laminar MHD flow near forward stagnation point of an impulsively rotating and translating sphere in presence of buoyancy forces are found by Dinarvand et al. [27]. Chamkha et al. [28] studied the unsteady MHD flow of mixed convection fluid with heat and mass transfer. Ram et al. [29] analyzed the influence of phase difference between highly oscillating magnetic field and magnetization on the unsteady ferro fluid flow due to a rotating disk. Nadeem et al. [30] worked out on the analytical treatment of unsteady mixed convection MHD flow on a rotating cone in a rotating frame.

Takhar et al. [31] examined the unsteady MHD flow of an ambient fluid with heat transfer over a rotating disk, present study aims at extending aforesaid work to incorporate the couple stress effects with magnetic field and heat transfer analysis is also taken into account. However, to the best of our knowledge, no attempt is available in the literature regarding unsteady MHD flow of couple stress fluid over a rotating disk. In this note, an investigation has been carried out to analyze the unsteady flow and heat transfer of couple stress fluid with the effect of transverse magnetic field on an infinite rotating disk along 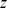-axis. The governing time dependent momentum and energy partial differential equations are transformed into a system of ordinary differential equations by using suitable similarity transformations. These ODEs are then solved numerically by employing a Runge-Kutta technique coupled with shooting method. The variations in the flow field, heat transfer and pressure of the fluid due to pertinent parameters are obtained through graphs and discussed thoroughly.

## Problem Formulation

**Figure 1:**
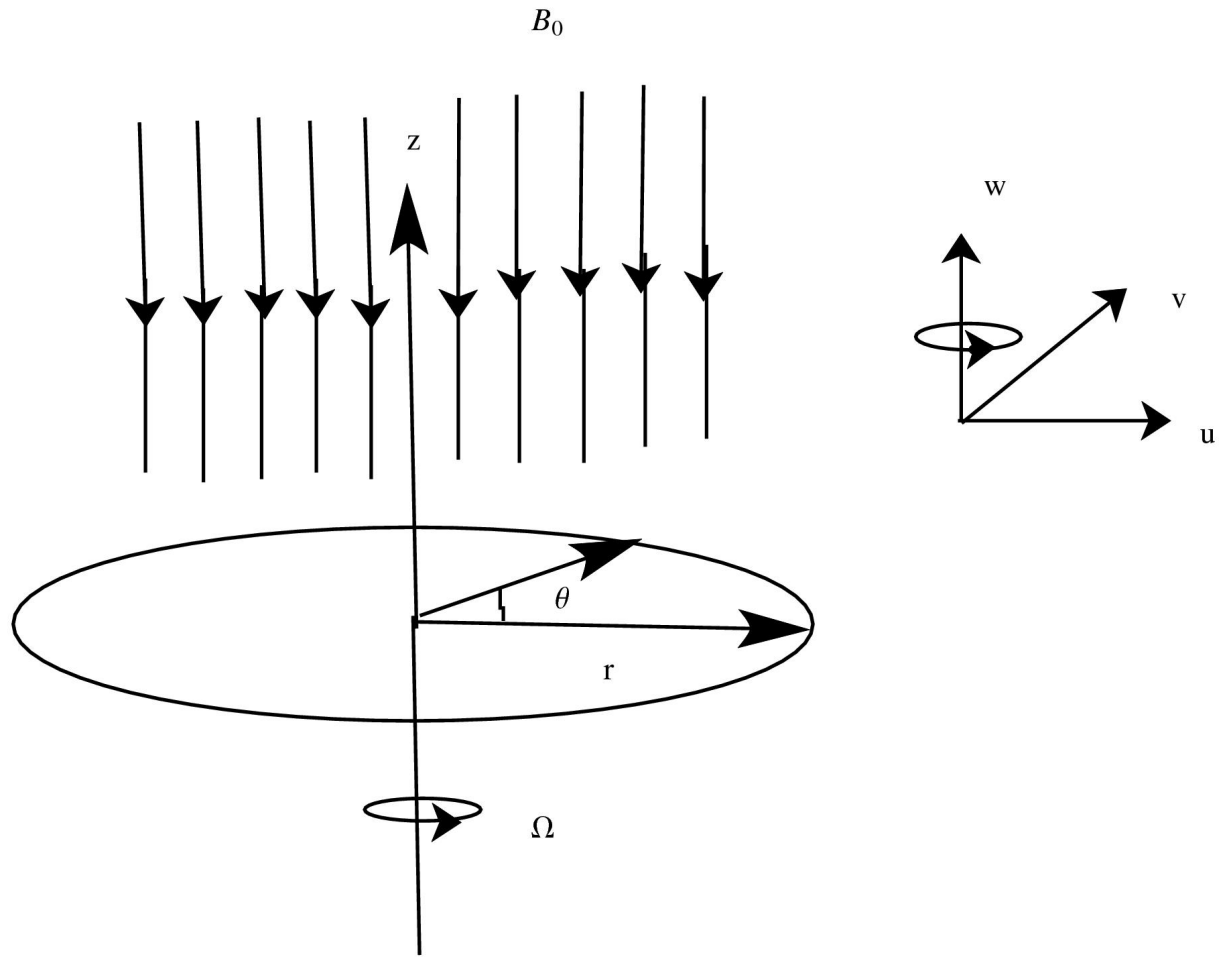
Coordinates system and flow configuration.

Consider the three dimensional, unsteady, laminar, incompressible, MHD flow and heat transfer of couple stress fluid over a rotating disk which rotates about the 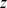-axis with angular velocity 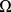 in a cylindrical polar coordinate system 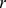, 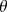 and 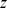. The 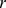-axis is in the direction of radius of disk, and the applied uniform magnetic field 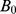 is in the direction of 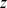-axis, normal to the surface of disk. As the flow is axisymmetric therefore radial, tangential and axial components of velocity 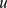, 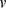, 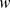, temperature 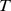 and pressure 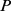 are independent of 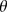. The induced magnetic field can be neglected in comparison with the applied magnetic field by considering the magnetic Reynolds number 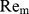 much less than 1. Fig. 1 represents the flow description and geometrical configuration of the physical model.

The governing partial differential equations are
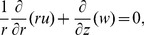
(1)




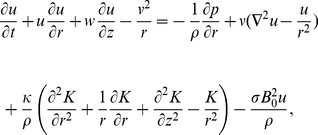
(2)




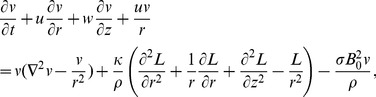
(3)




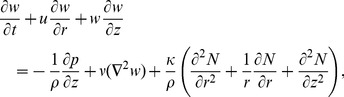
(4)




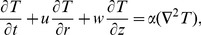
(5)where 
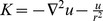
, 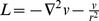, 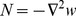, 
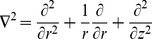
.

The boundary conditions are
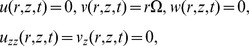





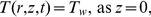
(6)




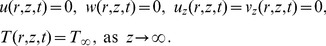
(7)


Here, 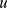, 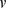 and 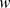 are the components of velocity along the 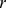, 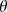 and 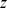 directions respectively, 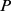 and 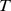 are the pressure and temperature, respectively. 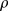, 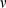 and 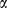 are density, viscosity and thermal diffusivity, respectively. 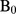 is the applied magnetic field, 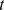 is the fixed time and 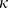 is the couple stress parameter respectively.

Using similarity transformations
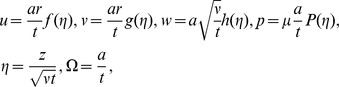





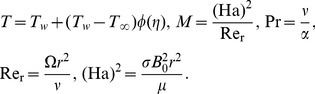
(8)


The equation of continuity yields the relation
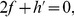
(9)


And Eqs. 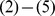, after using Eqs. (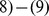 reduce to
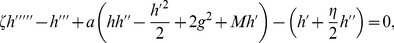
(10)




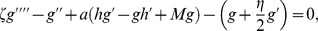
(11)




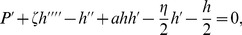
(12)




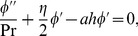
(13)


with boundary conditions



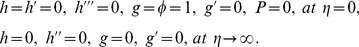
(14)


Here 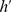, 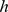 and 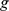 are the radial, axial and tangential components of the dimensionless velocity, 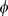 and 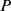 are the dimensionless temperature and pressure of the flow, 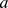 is the dimensionless unsteadiness parameter which measures the decrease or increase in the angular velocity 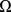, 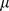 is the coefficient of viscosity, 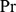 is the Prandtl number, 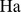 is the Hartmann number, 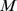 is the magnetic parameter, 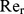 is the Reynolds number with respect to radius respectively. The dimensionless couple stress parameter 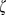 depends on time 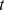, the Eqs.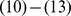 are not pure similarity equations, to avoid ambiguity, 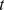 has been fixed which led the above equation to approximate similarity. Therefore 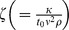 is valid approximation. And the solutions obtained here are approximations to solutions of the original nonlinear PDEs.

The shear stress of the fluid in the radial and axial directions can be found as
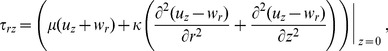
(15)and



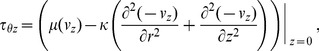
(16)Hence the local skin friction coefficients are given by
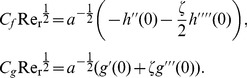
(17)


The heat transfer coefficient in terms of the Nusselt 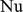 number can be expressed as
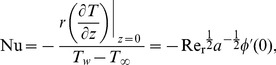
(18)


We use the result 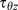 with a radial strip integration to find the total torque required to turn a disk of radius 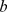.
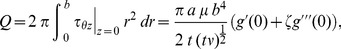
(19)


The dimensionless moment coefficient for a disk of radius 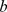 can be defined as
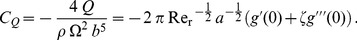
(20)


The system of Eqs. 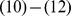 have been modeled the unsteady flow of a couple stress fluid over a rotating disk. The case when there is no body couples or rotation in the fluid, we set the couple stress parameter 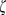 equal to zero in the governing system, which results in the following form
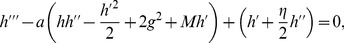
(21)




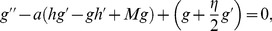
(22)




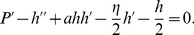
(23)


It can be noticed that the Eqs. 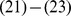 represents the model of unsteady flow of an electrically conducting viscous fluid which was considered and studied by Takhar et. al. [31].

## Numerical Procedure

The resulting highly nonlinear system of ODEs 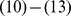 with boundary conditions 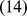 are solved numerically using fourth-order Runge-Kutta integration scheme with shooting technique for fixed time 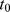. In shooting method, the resulting system of nonlinear ordinary differential equations is transformed to a set of simultaneous first order differential equations. A systematic guessing of initial conditions and numerical integration as an initial value problem has been done until the desired degree of accuracy has obtained. We have relied on the computational software MATHEMATICA package to solve the considered problem. Influences of all relevant parameters are presented graphically.

## Results and Discussion

**Figure 2:**
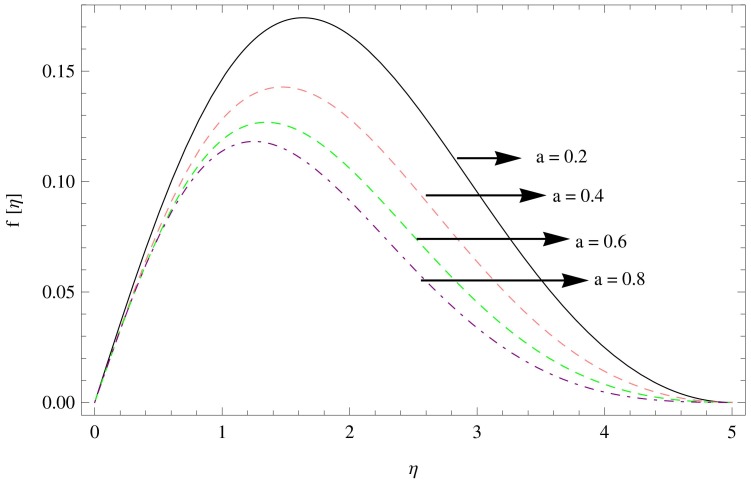
The radial velocity field for 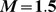, 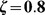.

**Figure 3:**
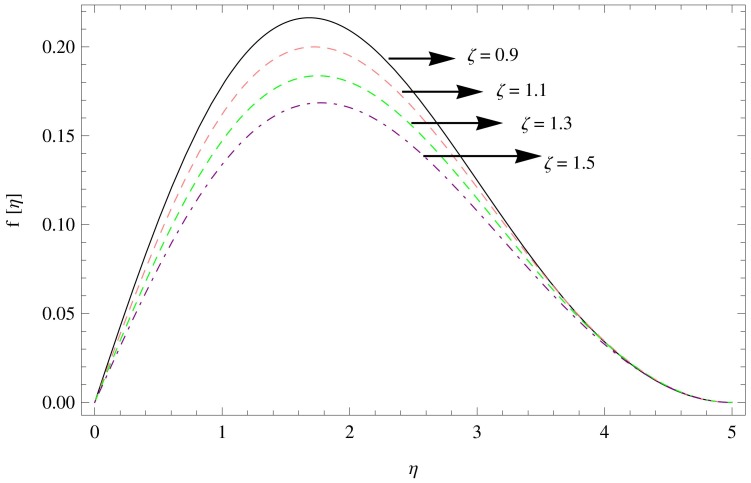
The radial velocity field for 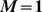, 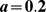.

**Figure 4:**
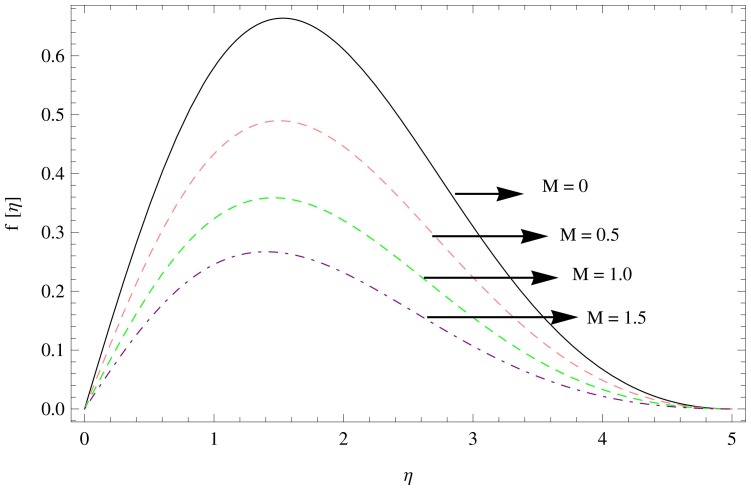
The radial velocity field for 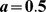, 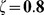.

**Figure 5:**
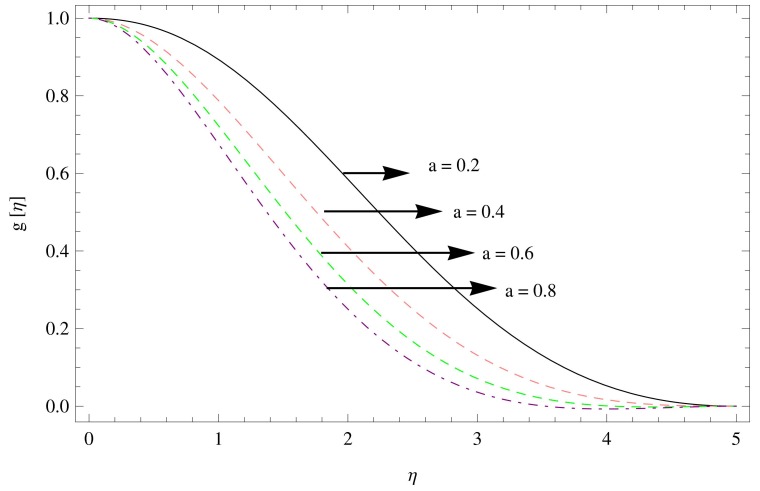
The azimuthal velocity field for 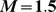, 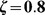.

**Figure 6:**
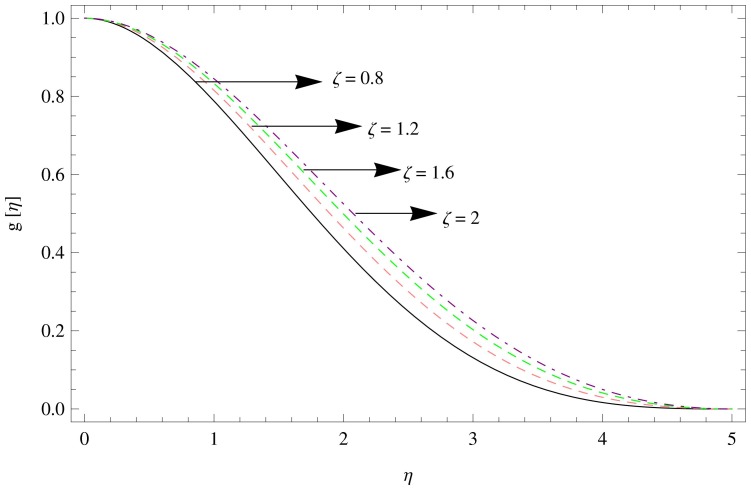
The azimuthal velocity field for 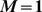, 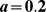.

**Figure 7:**
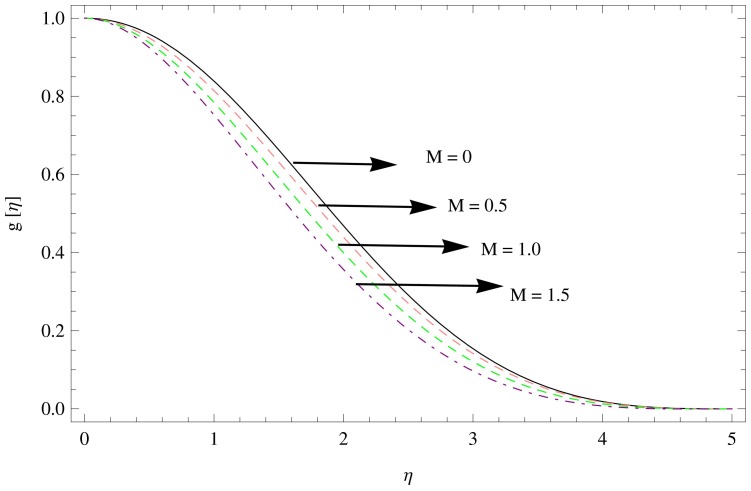
The azimuthal velocity field for 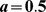, 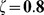.

**Figure 8:**
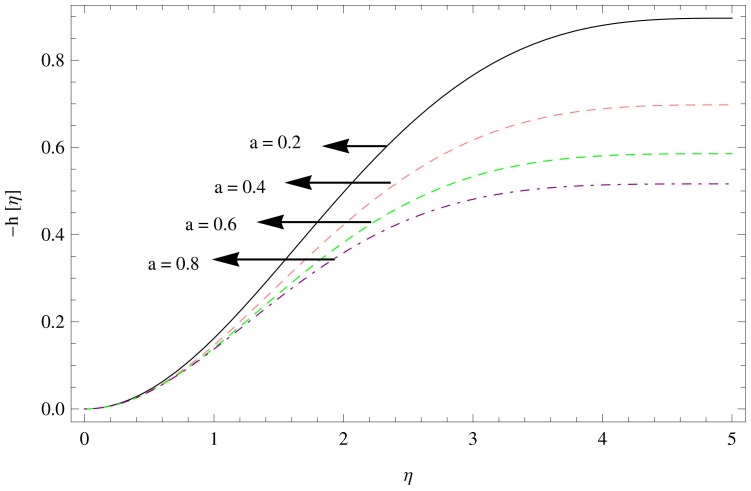
The axial velocity field for 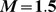, 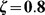.

**Figure 9:**
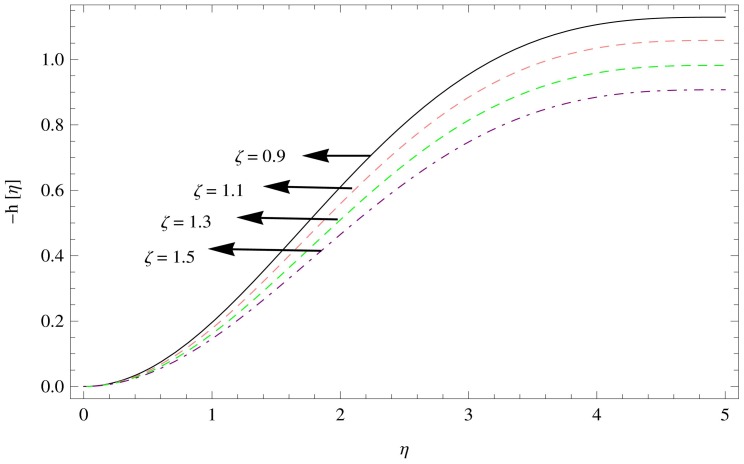
The axial velocity field for 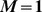, 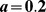.

**Figure 10:**
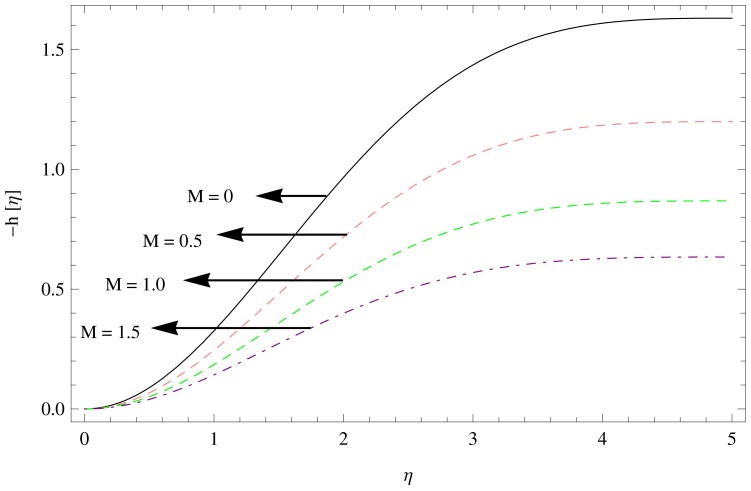
The axial velocity field for 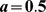, 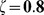.

**Figure 11:**
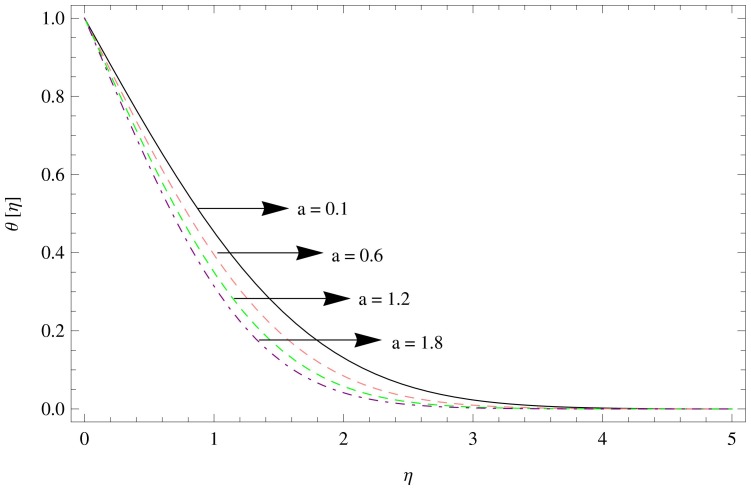
The temperature gradient for 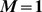, 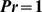, 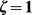.

**Figure 12:**
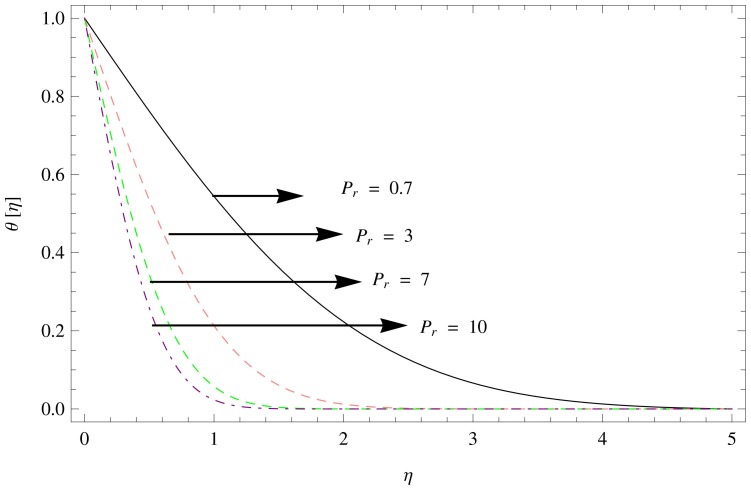
The temperature gradient for 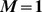, 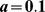, 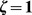.

**Figure 13:**
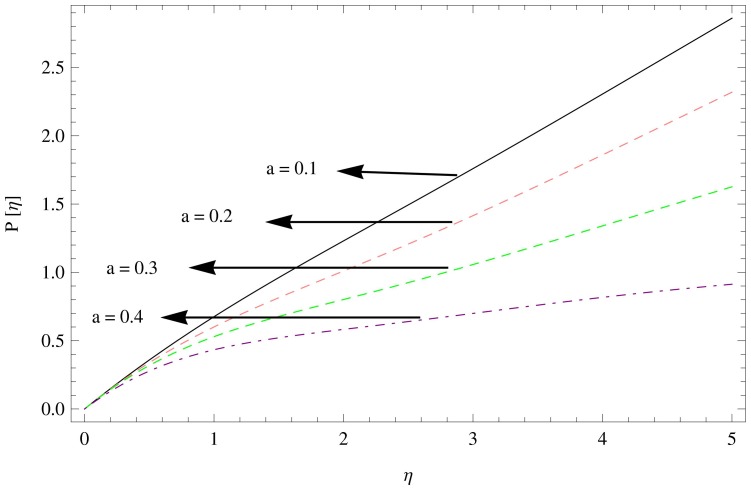
The pressure gradient for 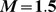, 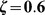.

**Figure 14:**
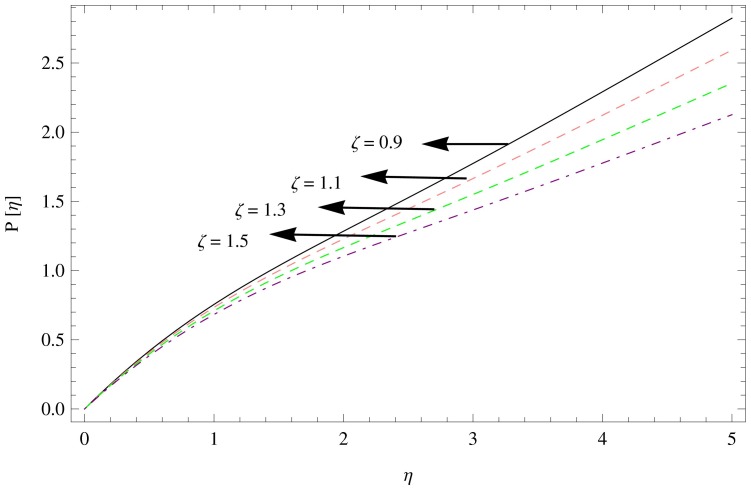
The pressure gradient for 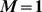, 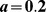.

**Figure 15:**
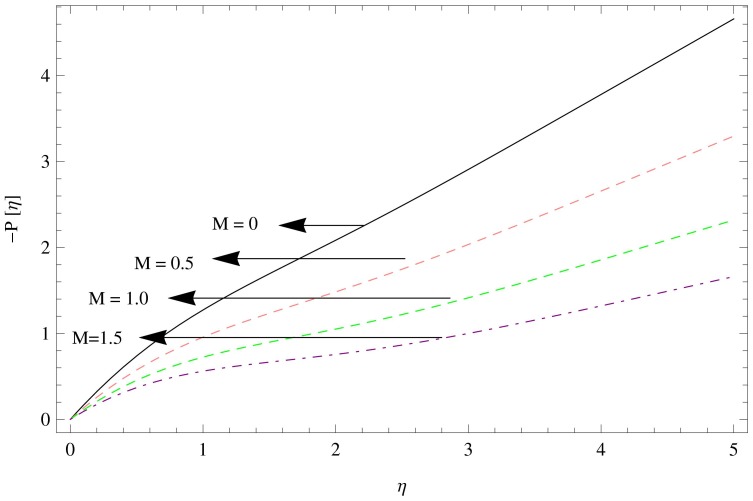
The pressure gradient for 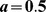, 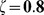.

The numerical solution of the problem has been computed via fourth order Runge-Kutta method based on shooting technique. The effect of various physical parameters namely the unsteadiness parameters 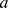, magnetic parameter 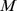, couple stress parameter 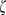 and Prandtl number 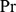 have been examined to measure the variations in the angular velocity, in the velocity field, in the temperature gradient and in the pressure distribution of the fluid (see Figs. 2–15). The changes in radial velocity 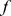 for the variations in the parameters 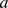, 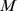 and 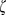 are displayed in Figs. 2–4. It can be seen from Fig. 2 that the unsteadiness parameter 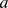 decreases the radial velocity 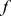, the stress due to the micro rotation particles in the fluid also weakens the motion of the couple stress fluid as increase in the couple stress parameter 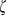 decreases the radial velocity (see Fig. 3), whereas Fig. 4 shows that the increase in magnetic parameter 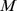 results in the decline of velocity function in radial direction which indicates that the inhibiting Lorentz forces opposes the flow in this direction.

The effects of tangential velocity 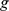 as a function of 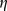 for 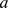, 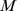 and 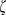 are shown in Figs. 5–7. The decline in the tangential velocity component can be observed with the increase in unsteadiness parameter 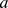 as shown in Fig. 5. An increase in the tangential flow over the disk is observed with the increment in the couple stress parameter 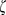 increasing the boundary wall thickness (see Fig. 6). Since the magnetic force is in the direction normal to the disk, it opposes the tangential flow velocity, therefore the tangential velocity is lowered everywhere. Thus the effect of the Lorentz forces leads to decelerate the azimuthal velocity with increase in 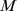. (see Fig. 7).

Figs. 8–10 shows the effect of aforesaid parameters on axial velocity 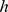 as a function of 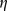. The axial velocity field behaves in a similar way to the radial velocity field. Flow field is reduced in the axial direction with the enhancement in the unsteadiness parameter 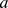 (as shown in Fig. 8), Fig. 9 shows the effects of increasing couple stress parameter which brings unfavorable change in the axial velocity component. The effect of magnetic parameter can be observed in Fig. 10 which displays the fall in the axial velocity component of couple stress fluid flow with the increase in magnetic parameter 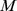.

In Figs. 11–12, the effect of temperature gradient 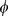 as a function of 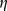 on the different values of 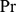 and 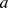 are observed. Increasing 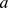 results in decline in the temperature gradient (Fig. 11), while the similar behavior is observed with increasing 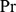 (Fig. 12).

Figs. 13–15 describes the outcome of the pressure distribution against 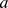, 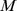 and 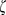. These figures depicts the inverse relation between pressure distribution and the unsteadiness parameter, magnetic parameter and couple stress parameter, indicating an increase in above mentioned parameters led the decreasing behavior of the pressure distribution.

## Limitations of the Study and Future Recommendations

It is imperative to enlighten diverse limitations of this research. This discussion aims at assisting readers to comprehend this study, which ultimately, provide an opportunity to extend the current research. The following assumptions and limitations are considered.

- Flow is unsteady, incompressible and laminar.
- Flow is three dimensional and rotating over a disk about the 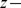 axis.
- A uniform magnetic field is applied in the direction normal to the surface of the disk which is assumed unchanging with a small magnetic Reynolds number 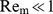.
- It is assumed that the effect of dissipation in the energy equation is negligible.

In this study we attempt to solve governing equations numerically for a fixed value of 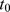 (i.e., at a fixed time ) since 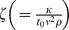. It is interesting to note that for large time the higher derivatives in equations 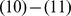 are vanished as 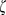 tends to zero. This leads the case of degenerate limit and difficult to solve with the presented technique, so a different approach should be sought such as singular perturbation theory to solve the problem for the large-time limit. It is worth observing that, on considering the aforesaid limitations for future research, it will turn out to be a demanding task for the researchers regardless of their particular interests in this area. For the model like couple stress fluid, an extensive range of problems can be defined for both theoretical and experimental aspects, i.e. flow over an off centered disk, between two coaxial disk, due to rotating sphere, over an inclined disk etc. So, fascinated by the diverse applications of non-Newtonian fluids, the present work can be extended to other non-Newtonian fluids model by employing a variety of numerical methods.

## Conclusions

In this paper, the response of unsteady MHD flow of couple stress fluid over a rotating disk is discussed. The nonlinear differential equations have been solved numerically using Runge-Kutta method and shooting technique. The numerical formulas for radial and tangential shear stress, heat transfer coefficient in term of Nusselt number and dimensionless moment coefficient are presented. The influence of various physical parameters and couple stress parameter on the velocity field, temperature and pressure distributions are all examined numerically and discussed graphically. The following results can be drawn:

- The unsteadiness parameter decelerates the flow and temperature field over the rotating disk.
- The presence of couple stresses in the fluid enhances the fluid motion in tangential direction and weakens in radial and axial directions.
- The magnetic field opposes the fluid velocity in all directions i.e. radial, tangential and axial.
- There is a significant reduction in the temperature field due to an increase in Prandtl number and unsteadiness parameter.
- Pressure gradient decline while unsteadiness parameter, magnetic parameter and couple stress parameter increases.
